# Anthelmintic Activity of Hydroethanolic Extract of *Senegalia senegal* (L.) Britton (Fabaceae) on the Small Ruminant Parasite *Haemonchus contortus*

**DOI:** 10.1155/japr/8782416

**Published:** 2025-05-28

**Authors:** Denis Danwe Djambo, André Perfusion Amang, Mathieu Djongra, Dieudonné Ndjonka

**Affiliations:** ^1^Department of Biological Sciences, Faculty of Sciences, University of Maroua, Maroua, Cameroon; ^2^Department of Biological Sciences, Faculty of Sciences, University of Ngaoundere, Ngaoundere, Cameroon

**Keywords:** anthelmintic, *Haemonchus contortus*, *Senegalia senegal*, small ruminants

## Abstract

Anthelmintics used to control haemonchosis in small ruminants have several limitations; the most notable of which are resistance and reinfestation. The aim of this study was to investigate the anthelmintic effect of the hydroethanolic extract of *Senegalia senegal* (HESS) against the parasite *Haemonchus contortus*. The hydroethanolic extract was tested in vitro on the various developmental stages of *H. contortus* and in vivo in goats infested by this parasite. The acute toxicity of HESS was assessed in mice according to Organisation for Economic Cooperation and Development (OECD) Line 425. HESS inhibited egg hatching with LC_50_ values = 1.51 mg/mL and LC_90_ = 2.57 mg/mL; this extract was also lethal with LC_50_ values = 1.25 mg/mL and LC_90_ = 1.99 mg/mL on Stage 3 larvae and LC_50_ = 1.28 mg/mL and LC_90_ = 1.04 mg/mL on adult females; HESS at a dose of 500 mg/kg deparasitized goats, inducing a 91.27% reduction in fecal egg excretion, a 94.73% reduction in parasite load, and a 57.65% reduction in female fertility. The secondary metabolites present in the extract were phenolic compounds, of which tannins (243.12 ± 0.76 mg ECA/gDM) accounted for the majority. HESS at a dose of 2000 mg/kg did not induce any clinical signs of toxicity or mortality in mice. These results would justify the traditional use of *S. senegal* to control the gastrointestinal strongyle, *H. contortus.*

## 1. Introduction

Small ruminants are widely distributed throughout the world and play a major role in providing income for some rural families through the marketing of livestock products such as milk, meat, hides, and wool [1]. In the livestock sector, small ruminants are threatened by numerous diseases caused by gastrointestinal strongyles, in particular haemonchosis, a disease caused by a parasite called *Haemonchus contortus*. This species is generally blamed for production losses in small ruminants [2].

The socioeconomic impact of gastrointestinal parasitosis is characterized by major animal losses due to helminthiasis prevalence rates of up to 98% in some regions [2]. The global prevalence of haemonchosis is 80% [3] with over 50% of cases in Africa [4], with the highest rates (90%) recorded in Kenya and Niger [5]. In northern Cameroon, the prevalence rate is 75% [6].

The use of anthelmintic molecules to treat gastrointestinal strongyles has many limitations, such as resistance to anthelmintics [3], slow elimination by the host [1], and the toxicity of these synthetic products. In addition, the low level of medical coverage and dependence on external supplies encourages their fraudulent reproduction [1]. Furthermore, the inaccessibility of veterinary products to small-scale livestock farmers in developing countries due to poverty [7] also constitutes a limitation. This highlights the need to look for alternative solutions to treat haemonchosis effectively, with few side effects, easy access, and low toxicity.

African flora abounds in plants with medicinal properties that can be exploited. Several authors have reported the anthelmintic effect of plants such as *Vitex thomasii* [8], *Senna rugose* [9], *Fumaria parviflora* [10], and *Vernonia amygdalina* [11]. *Senegalia senegal* is a deciduous shrub 2–6 mm high, up to 15 mm, and is typical of the South African regions from Senegal throughout the Sahel to Namibia, Angola, East Africa from Somalia and Saharan Africa, namely, Mali, Senegal, Chad, Egypt, Sudan, and Cameroon [12]. It is a Sahelian species of dry forests, wooded steppes, from sea level to around 600 m [12]. The leaves, bark, and gum are used as astringents to treat colds, hemorrhage, diarrhea, and helminths [12].

In order to verify the therapeutic effect of *S. senegal*, the objective was to evaluate the anthelmintic properties of the hydroethanolic extract of *S. senegal* (HESS) on the small ruminant parasite *H. contortus.*

## 2. Materials and Methods

### 2.1. Harvesting and Preparation of *S. senegal* Extract

Five kilograms of leaves was harvested in the Dolla locality (12°33⁣′0.472⁣^″^N, 10°17⁣′0.286⁣^″^E) in Garoua in the North Cameroon Region. They were harvested in March, dried for 2 weeks in the shade at room temperature (37°C), and ground to a powder (2000 g).

To obtain HESS, 1500 g of leaf powder was macerated in 15 L of 70% ethanol for 48 h at room temperature. After 48 h, the solutions were centrifuged at 3500 rpm for 10 min. The supernatant was then recovered and filtered using filter paper to collect the filtrate. The filtrate obtained was placed for a week in an oven set at 40°C. The extract obtained was weighed [13].

### 2.2. Collection of Adult Worms

Adult *H. contortus* worms were collected from the abomasum of freshly slaughtered goats and sheep at the Bantai market (Ngaoundere, Cameroon). The worms collected were examined under a dissecting microscope to identify adult female *H. contortus.* The worms were maintained in saline phosphate buffer at room temperature and used for anthelmintic tests.

### 2.3. Collection of *H. contortus* Eggs

*H. contortus* eggs were obtained using the technique described by [14] with a few modifications. Pregnant *H. contortus* females were placed in a plate containing 500 *μ*L of phosphate-buffered saline (PBS) and incubated at 37°C for 6 h. The adult females were removed and the eggs were collected after centrifugation for 5 min.

### 2.4. Harvesting Stage 3 *H. contortus* Larvae

Stage 3 larvae were obtained by coculture of eggs from gravid females, which were crushed using a mortar in order to release the eggs [15]. Stage 3 (L3) larvae from the development of these eggs were concentrated using the Baermann device. The previously dewormed goat was infested with approximately 4500 L3 by gavage using an esophageal tube. After 21 days, 15 g of feces was taken directly from the rectum of the reservoir goat and coprocultured for 8 days. The L3 were again concentrated using the Baermann device.

### 2.5. Chemical Materials

The chemical materials used throughout this study were mainly supplied by the Sigma Aldrich Laboratory in Germany. This material consists of PBS (pH 7.4). This is a physiological medium used to rinse and store *H. contortus* worms after isolation. Dimethyl sulfoxide (DMSO) was used to dissolve extracts and conventional drugs (levamisole).

### 2.6. Obtaining and Allocating Experimental Goats

This work was carried out in accordance with the Animal Ethical Committee of the Ngaoundere Regional Delegation of Livestock, Fisheries and Animal Industries Authority, Cameroon, Number 067/23/L/RA/DREPIA/A.

Twenty-four (24) goats, regardless of sex, were used for the in vivo tests. They were divided into six batches of four animals, including two control batches (normal and negative), one positive control batch, and three test batches (Table 1). The animals were purchased from breeders in the Benoue Department (North Region/Cameroon). All goats were tied to the neck with different-colored ropes and numbered buckles for identification purposes. They were then acclimatized in the animal house of the Pitoa Zootechnical Center for 1 week. During this period, the animals were simultaneously treated orally with levamisole (5 mg/kg) and tylosin 200% (10 mg/kg). During the experiment, the animals were kept in confinement in a pen. They were fed daily with ad libitum forage (*Pennisetum* sp.) (disinfected by washing with bleach 24 h beforehand) and borehole water.

### 2.7. Preparation of Extract Concentrations Administered

The concentrations of HESS solutions corresponding to the doses (125, 250, and 500 mg/kg) were calculated according to the following formula:

 $$\begin{array}{c} C=\frac{D\times M}{V\times 1000} \end{array}$$

where *C* is the concentration of extract to be administered; *D* is the dose of extract; *M* is the mass of animal; *V* is the volume to be administered to animals. The control batches (normal and negative) received distilled water, the positive control batch received a single dose of levamisole (5 mg/kg), and the other three test batches received HESS at doses of 125, 250, and 500 mg/kg. The extract was administered twice daily (morning and evening) for 3 days, while levamisole was administered as a single dose. All substances were administered orally via an esophageal tube.

### 2.8. Qualitative Phytochemical Screening of HESS

The phytochemical compounds present in HESS were highlighted by a number of characteristic tests.

#### 2.8.1. Detection of Polyphenols

The detection of polyphenols in HESS was carried out according to the method described by [16], using the ferric chloride (FeCl_3_) test. One milliliter of the extract was prepared to 0.1 mg / mL and then introduced into the previously cleaned glass tube. Four drops of 10% FeCl_3_ in methanolic solution were added to the tube. The appearance of a green-colored precipitate indicated the presence of polyphenols.

#### 2.8.2. Identification of Tannins

The presence of tannins in HESS was assessed by the FeCl_3_ test following the method described by [16]. One milliliter of the extract was prepared to 0.1 mg /mL and then introduced into the previously cleaned glass tube. Four drops of 10% FeCl_3_ in methanolic solution were added. The appearance of a greenish precipitate indicated the presence of tannins in the solution.

#### 2.8.3. Identification of Flavonoids

The presence of flavonoids in HESS was assessed using the Wilstater test described by the method of [16]. One milliliter of the extract was prepared to 0.1 mg /̸mL and then introduced into a tube. Three magnesium chips and 1-mL saturated hydrochloric acid were added in turn to the preparation. An orange coloration indicated the presence of flavones, a red coloration indicated flavonols, and a violet coloration indicated the presence of flavonones.

#### 2.8.4. Identification of Anthraquinones

Anthraquinone detection was carried out according to the protocol described by [16]. One milliliter of extract prepared at 0.1 mg /̸mL was introduced into a tube, and then, 2 mL of a mixture (petroleum ether /chloroform) and 2 mL of 10% NaOH were added to the preparation. A red coloration indicated the presence of anthraquinones.

#### 2.8.5. Identification of Alkaloids

The presence of alkaloids in HESS was determined by the Dragendorff test described by [17]. One milliliter of the evaporation residue (extract) was prepared at 0.1 mg /̸mL in a tube. One milliliter of 5% hydrochloric acid and three drops of Dragendorff's reagent were added, and a white or orange precipitate indicated the presence of alkaloids.

#### 2.8.6. Detection of Triterpenes

The Liebermann–Burchard test was used to detect triterpenes in our extracts. Following the protocol described by [16], 1 mL of extract was prepared at 0.1 mg /̸mL in a tube. One-milliliter chloroform, 1-mL concentrated H_2_SO_4_, and 1-mL acetic anhydride were added in turn. Purple coloration denoted the presence of triterpenes.

#### 2.8.7. Identification of Saponins

The presence of saponins in HESS was highlighted by the method of [16]. One milliliter of the extract was prepared at 0.1 mg /̸mL in a tube, 4-mL distilled water was added, and the mixture was shaken vigorously for 5 min. A persistent foam confirmed the presence of saponins.

#### 2.8.8. Quantitative Phytochemical Screening of HESS

Quantification of these groups present in the extract was carried out using the method described by [18] for total polyphenols and [19] for flavonoids and tannins.

### 2.9. HESS In Vitro Nematotoxic Test on the Different Stages of *H. contortus*

#### 2.9.1. Concentration Screening

To determine the concentration of nematicidal tests on all stages of *H. contortus* development, a calibration test was carried out on the most effective part of *S. senegal* at two concentrations: 1 and 5 mg/mL. The minimum concentration producing the greatest effect during a time slot of observation was used for further work.

#### 2.9.2. Testing the Anthelmintic Effects of HESS on *H. contortus* Eggs

To assess the hatching rate, the “egg hatch inhibition test,” which measures the ovicidal activity of anthelmintics, was used. This test was carried out using the method described by [20], which consists of incubating 50 strongyle eggs at a temperature of 37°C for 24–48 h at concentrations (0.50, 1, 1.50, 2, and 2.50 mg/mL). Each petri dish received 500 *μ*L of HESS and levamisole (positive control) and 500 *μ*L of PBS (negative control). All operations were repeated three times. Petri dishes were incubated at 37°C for 48 h. A volume of 20 *μ*L of formalin was added to each petri dish to stop the development of the eggs. After the required time, the eggs that had not hatched were counted and the percentage of HESS inhibition was determined by calculating the egg hatching inhibition rate (EHI) using the formula:

 $$\begin{array}{c} \text{EHI}\left(\%\right)=\left[\frac{\text{number of eggs}}{\text{number of L}1\text{ larvae}+\text{number of eggs}}\right]\times 100. \end{array}$$

#### 2.9.3. Test of the Anthelmintic Effects of HESS on Stage 3 Larvae of *H. contortus*

The “L3 larval paralysis test” was carried out to test the paralysis effect of levamisole and HESS. It was developed by [21]. In order to improve contact between the larvae and the extract, they were unsheathed by soaking in a 1.5% sodium hypochlorite (NaOCl) solution for 15 min. Larvae (L3) obtained by coproculture were incubated at 25°C at concentrations of 0.50, 1, 1.50, 2, and 2.50 mg/mL. Each petri dish received 500 *μ*L of HESS and levamisole (positive control) and 500 *μ*L of PBS (negative control). The petri dishes were placed at room temperature for 24 h and then for 48 h. The larvae were classified by observing their mobility. All concentrations were repeated three times, and the larval mortality rate was calculated using the formula of [15]:

 $$\begin{array}{c} \text{Mortality rate}\left(\%\right)=\frac{\text{number of dead L}3\text{ larvae}}{\text{total number of L}3\text{ larvae in culture}}\times 100. \end{array}$$

#### 2.9.4. HESS Anthelmintic Test on Adult Female *H. contortus* Worms

For the nematotoxic test itself, adult female worms were cultured in two 24-well plates. For this purpose, 18 wells of each plate each received a volume of 500 *μ*L of plant extract and levamisole at different concentrations (0.20, 0.40, 0.60, 0.80, and 1 mg/mL), respectively (one worm per well) and 500 *μ*L of PBS (negative control). Three trials were carried out for each concentration series. The worms were incubated at 37°C, and mortality was assessed after 24 h after every 6 h of time.

Worm mortality was determined using the following formula:

 $$\begin{array}{c} \text{Mortality rate}\left(\%\right)=\frac{\text{number of dead worms}}{\text{total number of worms on departure}}\times 100. \end{array}$$

### 2.10. In Vivo Nematotoxic Test of HESS in Goats Infested With *H. contortus* Larvae L3

#### 2.10.1. Dose and Substance Administration Protocol by Group

Animals aged less than 1 year and weighing 10 ± 3 kg were divided into six batches of four animals without distinction of sex. Two control batches (normal and negative) received distilled water, one positive control batch received a single dose of levamisole (5 mg/kg) once, and three test batches received EHAS at doses of 125, 250, and 500 mg/kg. Extract was administered daily for 3 days. All substances were administered orally via an esophageal tube. Percentage reductions in fecal egg density, parasite load, hematocrit, and body mass were determined.

#### 2.10.2. Infestation of Goats With *H. contortus* Larvae L3

Each animal was then infested with approximately 4500 L3 larvae of *H. contortus* from the coproculture previously carried out. All infestations were carried out using an esophageal probe, which enables all the larvae to be deposited directly in the rumen. The operation was repeated three times for effective infestation. After 21 days, a quantitative infestation check was carried out to verify the level of infestation in the animals. Eggs per gram (EPGs) of fecal matter were quantified using the McMaster technique. This involves crushing 4 g of fecal matter taken directly from the rectum in 56 mL of a saturated sodium chloride solution (which allows the eggs contained in the fecal matter to float). After filtering the solution, the number of eggs counted was that contained in one hundredth of a gram of feces. The preparation was then examined under a microscope (Olympus) with a ×10 objective.

#### 2.10.3. Antemortem Evaluations

##### 2.10.3.1. Determination of the Percentage Reduction in Fecal Egg Excretion

Individual fecal samples were taken directly from the rectum of each animal before treatment (D22) and after treatment (D26, D29, D36, and D43). Coproscopies were taken to determine the number of EPG of feces:

 $$\begin{array}{c} \text{EPG}=\text{number of eggs in both compartments}\times 50. \end{array}$$

##### 2.10.3.2. Evaluation of the Rate of Reduction of Fecal Excretion of Eggs (RFE%)

The RFE% was calculated using the formula of [15]:

 $$\begin{array}{c} \text{RFE}\%=\frac{\text{initial EPG}-\text{EPG at time }t}{\text{initial EPG}}\times 100, \end{array}$$

with RFE% being the percentage of reduction of fecal egg excretion and EPG the egg per gram.

##### 2.10.3.3. Determination of Body Mass

Individual animals were weighed before treatment (D22) and at different times (D26, D29, D36, and D43) in order to assess weight trends in the different batches. The average daily weight gain (ADWG) was calculated using the following formula:

 $$\begin{array}{c} \text{ADWG}=\frac{Gt-Gi}{Gt} \end{array}$$

with ADWG being the average daily weight gain, *Gt* the weight gain at time *t*, and *Gi* the weight gain at initial time.

##### 2.10.3.4. Determination of Hematocrit Level

To assess the variation in hematocrit level before treatment (D22) and after treatment (D26, D29, D36, and D43), blood samples were taken in EDTA tubes from the jugular vein in the animal's neck. Once the blood had been collected in the EDTA tubes, the samples were placed in capillary hematocrit tubes and centrifuged for 5 min. The hematocrit level was read using an EKF Reaxtop hematocrit reader.

#### 2.10.4. Postmortem Assessment

##### 2.10.4.1. Determination of the Percentage Reduction in Parasite Load (RPL%)

To determine the RPL%, the animals were sacrificed and the abdominal cavity was opened. The digestive tract was isolated, and the sectioned abomasum was placed in a container containing 5 L of water. The abomasum was then opened longitudinally and the contents collected in the container. The inner wall of the abomasum was washed and gently scraped with a knife to remove the attached worms. The solution obtained was homogenized and then left to settle for 30 min. After homogenization, the worms were counted under a binocular loupe with a ×10 objective. The total number of worms was determined and the percentage reduction in the parasite load was calculated using the formula proposed by [22]:

 $$\begin{array}{c} \text{RPL}\%=\frac{\text{APICn}-\text{APITr }}{\text{APICn}}\times 100 \end{array}$$

where APICn is the average parasite intensity of batch negative control; APITr is the average parasite intensity of treated batches; RPL% is the percentage reduction in parasite load.

##### 2.10.4.2. Study of the Fecundity of *H. contortus* Females

The fecundity of the females was assessed by the number of eggs they contained in their uterus. Two-thirds of the females from each group were isolated and washed in a PBS solution and then individually introduced into a petri dish containing a 4% sodium hypochlorite solution until complete disintegration. The average number of eggs was determined using the formula below:

 $$\begin{array}{c} \text{ANE}=\frac{\text{total number of eggs}}{\text{total number of individuals}} \end{array}$$

with ANE being the average number of eggs per group.

### 2.11. HESS Acute Toxicity Test on Mice

The acute toxicity test was conducted in accordance with [23], which consists of testing HESS at a dose of 2000 mg/kg. The test was performed on six female mice. They were divided as follows: a control batch of three females given distilled water (10 ml/kg) and an experimental batch of three females given the single-dose extract (2000 mg/kg). Mortality and behaviors such as piloerection, lack of appetite, agitation, abdominal constriction, convulsion, motor difficulty, accelerated respiration, and somnolence were observed every 4 h on the first day and every day for 14 days after administration of the substances.

### 2.12. Statistical Analysis

The results obtained from the various tests on *H. contortus* were entered into various data analysis and processing software programs. Means, standard deviations, and curve plotting were carried out using GraphPad Prism Version 8.0 statistical analysis software. Log-probit determination of LC50 was performed using SPSS software Version 17.0. Data were analyzed using two-way ANOVA. Dunnett's nonparametric test was used for comparison of parameter variance. The difference between two variances was significant if *p* < 0.05.

## 3. Results

### 3.1. Qualitative and Quantitative Phytochemical Composition

The qualitative phytochemical analysis of HESS revealed the presence of tannins, phenolic compounds, and flavonoids. Quantitatively, HESS contains 109.82 ± 2.37 mg EAG/g DM of phenolic compounds, 4.62 ± 2.46 mg ER/g DM of flavonoids, and 243.12 ± 0.76 mg ECA/g DM of tannins.

### 3.2. In Vitro Anthelmintic Effects of HESS on the Different Stages of *H. contortus*

#### 3.2.1. In Vitro Anthelmintic Effects of HESS on *H. contortus* Eggs

**Figure 1:**
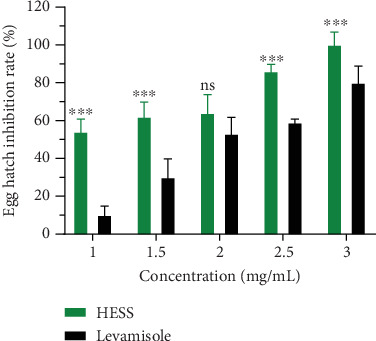
Diagram of variation in the rate of inhibition of *H. contortus* egg hatching as a function of HESS concentrations (milligram/milliliter) after 48-h incubation. Values are expressed as mean ± standard error of the mean. HESS: hydroethanolic extract of *S. senegal*; NS: not significant; ⁣^∗∗∗^*p* < 0.001, significant difference from positive control.

Figure 1 illustrates the evolution of the hatching inhibition rate of *H. contortus* eggs subjected to different concentrations of HESS after 48 h of incubation. It can be seen from this figure that the different treatments resulted in an increase in the average rate of inhibition of hatching of *H. contortus* eggs as a function of concentration. In fact, HESS at concentrations of 0.50, 1, 1.50, 2, and 2.50 mg/mL induced an anthelmintic activity by causing an increasing inhibition of egg hatching of 52.22%, 64.44%, 76%, and 86.66%, respectively; this reached the maximum rate of 100% at the concentration of 3 mg/mL.

#### 3.2.2. In Vitro Anthelmintic Effects of HESS on *H. contortus* Stage 3 Larvae

**Figure 2:**
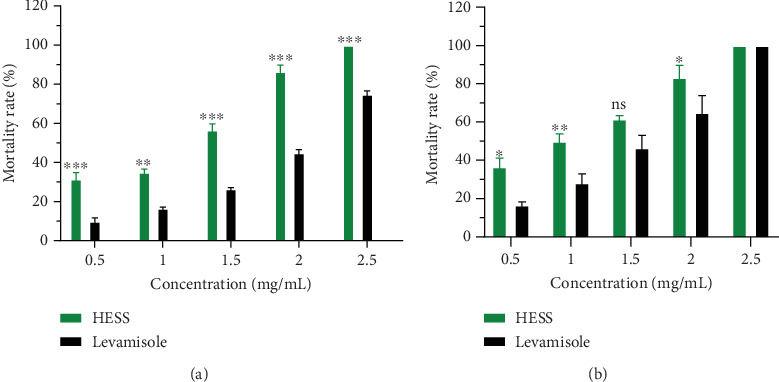
Variation in mortality rate (percentage) of *H. contortus* Stage 3 larvae as a function of HESS concentrations (milligram/milliliter) after 24 h (a) and 48 h (b) incubation. Values are expressed as mean ± standard error of the mean. HESS: hydroethanolic extract of *S. senegal*; NS: not significant; ⁣^∗^*p* < 0.05, ⁣^∗∗^*p* < 0.01, and ⁣^∗∗∗^*p* < 0.001, significant difference from positive control.

Figure 2 shows the mortality rate of Stage 3 larvae by HESS and levamisole as a function of concentration and time after 24 h (a) and 48 h (b) of incubation. This figure shows that HESS caused larval mortality for all the concentrations tested after 24 and 48 h of incubation. The highest concentration (2.50 mg/mL) caused 100% larval mortality after 24 h of exposure to EHAS. In contrast, levamisole induced 76.66% paralysis mortality in infesting larvae after 24 h incubation at the maximum concentration. Concentrations of 0.50, 1, 1.50, 2, and 2.50 mg/mL induced larval mortality of 31.66%, 33.33%, 56.66%, and 88.88%, respectively, for HESS and 10%, 26.66%, 45%, and 76.66% for levamisole after 24 h. After 48 h, the mortality rates were 36.66%, 50%, 61.33%, and 83.33% for HESS and 16.66%, 28.88%, 46.66%, and 65% for levamisole. The anthelmintic effect was greater with HESS after 24-h incubation.

#### 3.2.3. In Vitro Anthelmintic Effects of HESS on Adult Female *H. contortus* Worms

**Figure 3:**
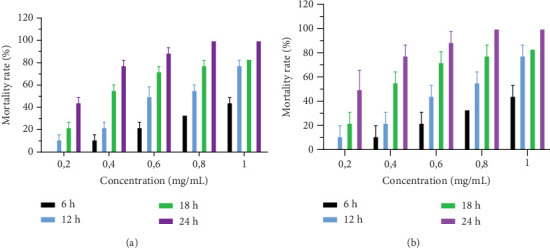
Variation in the mortality of adult female *H. contortus* as a function of the concentration (milligram/milliliter) in the presence of HESS (a) and levamisole (b) after 24-h incubation. Values are expressed as mean ± standard error of the mean. HESS: hydroethanolic extract of *S. senegal.*

After 24-h exposure to different concentrations of HESS, variations in the mortality rate of adult female *H. contortus* worms are shown in Figure 3. HESS induced mortality in adult worms in a concentration-dependent manner. This increasing variation in the mortality rate of adult *H. contortus* worms was 33.33% at 18 h and 83.33% at 24 h with HESS.

#### 3.2.4. Summary of HESS Lethal Concentration 50 and 90 Values for the Different Developmental Stages of *H. contortus*

Table 1 shows the LC_50_ and LC_90_ values of HESS tested on the stages of *H. contortus.* It can be seen that HESS has low LC_50_ and LC_90_ values compared with levamisole. The lethal concentrations determined for all the different tests of HESS carried out on the developmental stages of *H. contortus* revealed a remarkable efficacy compared to levamisole.

### 3.3. Deworming Effects of HESS in Treated Goats

#### 3.3.1. Effects of HESS on Fecal Egg Excretion

Data collected following administration of HESS were used to compile Table 2. Following administration of HESS and levamisole to experimental animals, the major result of this study in terms of EPG was a decrease in fecal excretion of *H. contortus* eggs in treated goats. Administration of HESS and levamisole to the animals resulted in a significant (*p* < 0.05) reduction in fecal excretion of parasite eggs throughout the experiment compared with the negative control. HESS induced a dose-dependent reduction in fecal egg excretion of 66.07%, 76.90%, and 91.27% at doses of 125, 250, and 500 mg/kg, respectively. Comparison of the values for reduction in fecal excretion at the end of observation revealed that there was no significant difference (*p* > 0.05) between the concentration values. However, at the maximum dose of 500 mg/kg, the reduction in egg excretion was significantly (*p* < 0.05) greater than that of the negative control.

#### 3.3.2. Effect of HESS on Hematocrit Levels in Goats

**Figure 4:**
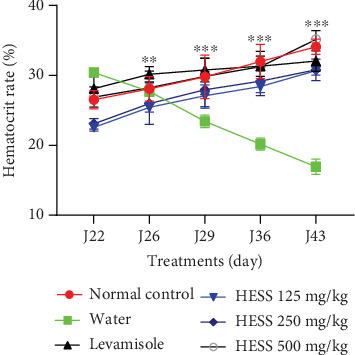
Variation in mean hematocrit levels in treated animals. *N* = 4: number of animals; values are expressed as mean ± standard error of the mean. HESS: hydroethanolic extract of *S. senegal*. ⁣^∗∗^*p* < 0.01 and ⁣^∗∗∗^*p* < 0.001 significant difference from negative control.

Figure 4 shows the hematocrit values before and after treatment of the experimental animals with HESS. The results of the mean hematocrit values presented in Figure 4 show a variation in the values of this parameter from one group to another. Administration of HESS to goats parasitized by *H. contortus* resulted in an increase in hematocrit from 22.57 ± 0.50% to 30.70 ± 0.63%, from 23.08 ± 0.74% to 30.08 ± 1.54%, and from 26.90 ± 1.45% to 35.15 ± 1.25%, respectively, at doses of 125, 250, and 500 mg/kg. Like HESS, levamisole also induced a significant (*p* < 0.05) increase in hematocrit from 28.10 ± 1.73% to 32.05 ± 1.47%. On the other hand, in the negative control group which received no treatment, a significant reduction in the mean hematocrit value was observed during the treatment period (*p* < 0.05).

#### 3.3.3. Effects of HESS on Goat Body Mass

Table 3 shows the mean values of the average gain in daily body mass of the goats treated with HESS. In general, there was a variation in mean body weight gain between batches. HESS induced a significant increase (*p* < 0.05) in the body mass gain of animals from 0.09 ± 0.01 to 0.25 ± 0.07, 0.09 ± 0.01 to 0.25 ± 0.04, and 0.03 ± 0.04 to 0.29 ± 0.03, respectively, at doses of 125, 250, and 500 mg/kg. Like HESS, levamisole induced a significant increase in animal body mass from 0.08 ± 0.00 to 0.23 ± 0.04 throughout the posttreatment period. However, in the negative control group that received no treatment, a significant decrease (*p* < 0.05) in body mass was observed.

#### 3.3.4. Effects of HESS on Parasite Load in Goats

Table 4 shows the effect of HESS and the RPL% in treated animals. It can be seen that the different treatments administered to the batches induced significant effects (*p* < 0.05) on the parasite load compared with the negative control batch. No significant difference was noted between the different treatment batches and the batch treated with levamisole. HESS resulted in a reduction in parasite load of 88.62%, 92.93%, and 94.73% at doses of 125, 250, and 500 mg/kg, respectively.

#### 3.3.5. Effects of HESS on the Fertility of *H. contortus* Females

**Figure 5:**
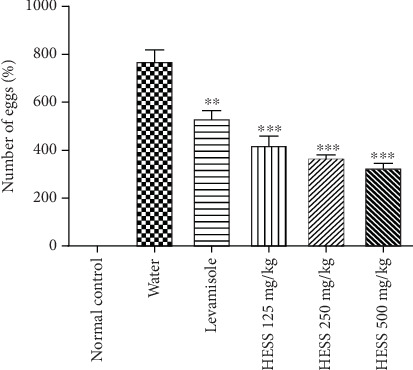
Variation in fertility of *H. contortus* after administration of different doses of HESS. *N* = 4: number of animals; values are expressed as mean ± standard error of the mean. HESS: hydroethanolic extract of *S. senegal*. ⁣^∗∗^*p* < 0.01 and ⁣^∗∗∗^*p* < 0.001 significant difference from negative control.

Figure 5 shows the evolution of the fertility of *H. contortus* females through the number of eggs. HESS induced a dose-dependent reduction in the fertility of *H. contortus* females (Figure 5). HESS significantly (*p* < 0.05) reduced the number of eggs in utero by 45.38%, 52.28%, and 57.65% at doses of 125, 250, and 500 mg/kg, respectively. On the other hand, in animals in the negative control group that received no treatment, a significant increase (*p* < 0.05) in the number of *H. contortus* eggs was observed throughout the study period.

### 3.4. Acute Toxicity Test in Mice

Observation of the animals in this study showed no change in general physical appearance or behavior and no mortality over the 14-day experimental period (Table 5).

#### 3.4.1. Changes in Body Weight in Mice After Administration of the Extract

**Figure 6:**
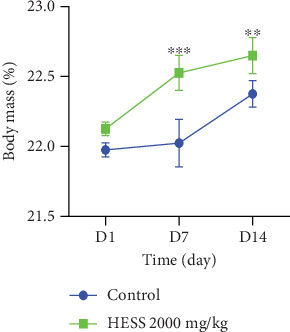
Variation in body mass in mice after administration of HESS. Values are expressed as mean ± standard error of the mean. HESS: hydroethanolic extract of *S. senegal*. ⁣^∗∗^*p* < 0.01 and ⁣^∗∗∗^*p* < 0.001 significant difference from control.

Oral administration of HESS at a dose of 2000 mg/kg to the animals induced a nonsignificant (*p* < 0.05) increase in the body mass of the animals in the different groups of the order of 0.36 and 0.27 g (Figure 6).

## 4. Discussion

The effects of secondary plant metabolites on gastrointestinal parasitism in small ruminants are increasingly being studied, especially as their consumption by livestock appears to represent an alternative method of controlling gastrointestinal nematodes. In this study, the in vivo and/or in vitro anthelmintic effects of HESS were evaluated on all stages of *H. contortus* development. In the in vitro test, HESS was brought into direct contact with the eggs, larvae, and adult females of the parasite in order to assess its inhibitory effect on egg hatching and/or its paralyzing effect on L3 and adult females. It was found that increasing concentrations of HESS resulted, on the one hand, in increasing inhibition of egg hatching and, on the other hand, in an increase in the rate of paralysis of L3 larvae and adult females of *H. contortus*. Generally speaking, at the highest concentration (3 mg/mL), the effects observed on the inhibition of egg hatching, on the paralysis of L3 larvae, and on adult female *H. contortus* were the most remarkable. Such an increase (in egg hatch inhibition, paralysis of L3 larvae, and adult females) as a function of HESS concentrations has also been observed in many previous in vitro studies [11, 24–26]. In this study, HESS was the most active in inhibiting the hatching of *H. contortus* eggs, showing a significant difference (*p* < 0.001) compared to levamisole at a concentration of 3 mg/mL. Several classes of secondary metabolites such as tannins, flavonoids, and phenolic compounds present in HESS are (taken individually or synergistically) endowed with anthelmintic properties [15]. According to [27], secondary metabolites of plant extracts such as tannins could diffuse more easily either to the outer surface of the eggshell and inhibit hatching or through the cuticle of the L3 and bind to the available free proteins, rendering them unusable as nutrients, leading to paralysis of larval and adult female *H. contortus.*

Throughout the in vivo experimentation, HESS was tested in goats infested with L3 of the gastrointestinal nematode *H. contortus* to assess the reduction in fecal egg excretion, hematocrit level, body mass, parasite load, and fertility of *H. contortus* females. Reference [8] showed that in goats, the presence of the gastrointestinal nematode *H. contortus* correlates significantly with the number of eggs present in feces, variation in hematocrit level, and body mass. This shows that HESS significantly (*p* < 0.001) reduced fecal egg excretion and the parasite load and increased hematocrit levels and body mass throughout the experiment in a dose-dependent manner. These observed effects would be due to the fact that HESS contains phytochemical compounds such as tannins, flavonoids, and phenolic compounds, which have effects on adult female *H. contortus* because these compounds act by paralyzing them. As a result, they reduce the number of females and prevent their fertility and the excretion of eggs in feces. According to [15], anthelmintic efficacy depends on the quantity and proportion of phytochemical compounds present in the plant. These results are similar to those obtained by [28] on the anthelmintic activity of *Fumaria parviflora* (Fumariaceae), who concluded from this study that the extract of this plant resulted in a dose- and time-dependent reduction in egg excretion and that the action of tannins and flavonoids, alone or in combination, could explain the observed reduction in EPG. The significant (*p* < 0.01) increase in hematocrit and body mass in our results suggests an anthelmintic effect of HESS on the nematode *H. contortus*, a hematophagous parasite capable of causing more or less severe, sometimes lethal anemia in its host. According to several authors [9, 29, 30], the administration of plants with a high tannin content leads to a reduction in the parasite load, as the tannins disrupt the functions or structures vital to the nematodes, in this case the integrity of the adult worm cuticle, a structure rich in proline and hydroxy-proline, thus favoring the high availability of proteins in the intestine, which corresponds to an additional supply of DPIFO (digestible proteins in the intestine of food origin). This stimulates the host's immune response, helping to boost its resistance and resilience in the face of parasitic aggression.

HESS showed no signs of toxicity or mortality in experimental animals at 2000 mg/kg. This observation would be in agreement with some authors [8, 26, 29] who have shown that the aqueous extract of *S. senegal* did not exhibit acute toxicity in Wistar rats. This significant increase in the mass of rats after administration of HESS at the maximum dose of 2000 mg/kg could explain the power of HESS to restore the triglyceride stock, thanks to the improvement in insulin secretion and glycemia observed by [8, 14, 23]. With an LD_50_ greater than 2000 mg/kg, this plant can be considered not acutely toxic according to the Globally Harmonized System of Classification of Toxic Substances.

## 5. Conclusion

This study demonstrated the in vitro and in vivo anthelmintic activity of *S. senegal* in small ruminants, in particular against the gastrointestinal nematode *H. contortus*. HESS demonstrated anthelmintic activity against the *H. contortus* parasite by inhibiting egg hatching, paralysis of infesting larvae, and mortality of adult *H. contortus* females. In addition, HESS also showed anthelmintic activity by reducing fecal excretion of *H. contortus* eggs, parasite load, and fertility of *H. contortus* females, thanks to the presence of phytoconstituents. HESS administered to mice produced no clinical signs of toxicity or mortality. In view of these results, it can be said that the use of this plant by farmers as an anthelmintic for small ruminants is justified and may represent an alternative for controlling gastrointestinal nematodes in small ruminants.

## Figures and Tables

**Table 1:** Lethal concentrations 50 and 90 of HESS on the different stages of *H. contortus.*

**Development stage**	**Lethal concentration 50 (mg/mL)**		**Lethal concentration 90 (mg/mL)**	
	**HESS**	**Levamisole**	**HESS**	**Levamisole**
Egg	1.51 ± 0.10	2.13 ± 0.11	2.57 ± 0.03	3.23 ± 0.02
Larvae (L3) at 24 h	1.25 ± 0.41	2.34 ± 0.03	1.99 ± 0.05	4.89 ± 0.07
Larvae (L3) at 48 h	1.34 ± 0.01	1.62 ± 0.01	2.18 ± 0.06	2.45 ± 0.03
Adult females	1.28 ± 0.03	1.86 ± 0.04	1.04 ± 0.01	2.13 ± 0.19

*Note:* Values are expressed as mean ± standard error of the mean.

Abbreviation: HESS, hydroethanolic extract of *S. senegal*.

**Table 2:** Change in mean density ± standard deviation of *H. contortus* egg and percentage reduction in fecal excretion (RFE%) after administration of HESS.

**Treatments**	**Pretreatment**	**Posttreatment**			
	**D**_**22**_ ** (** **n** = 4**)**	**D**_**26**_ ** (** **n** = 4**)**	**D**_**29**_ ** (** **n** = 4**)**	**D**_**36**_ ** (** **n** = 4**)**	**D**_**43**_ ** (** **n** = 4**)**
Normal control	0.00 ± 0.00	0.00 ± 0.00	0.00 ± 0.00	0.00 ± 0.00	0.00 ± 0.00
Water	1168 ± 13.04	1166.75 ± 14.08 (0.17%)	1171.5 ± 18.48 (−0.27%)	1173 ± 6.78 (−0.40%)	1336.5 ± 142.03 (−14.20%)
Levamisole	1811 ± 12.97	149.62 ± 22.45 (36.50%)	781.75 ± 96.73 (56.80%)	555 ± 61.21 (69.30%)	81.25 ± 23.32^∗∗∗^ (97.10%)
HESS 125 mg/kg	1109.50 ± 12.97	978.50 ± 122.86 (11.80%)	804.75 ± 139.08 (27.46%)	629.50 ± 96.65 (43.26%)	376.75 ± 50.58^∗∗∗^ (66.07%)
HESS 250 mg/kg	1117.75 ± 6.84	957 ± 122.86 (14.38%)	652.75 ± 97.88 (41.60%)	563 ± 122.78 (49.63%)	258 ± 54.47^∗∗∗^ (76.90%)
HESS 500 mg/kg	1241.21 ± 65.88	755.25 ± 125.88 (39.15%)	624.25 ± 92.71 (49.70%)	599.75 ± 81.78 (51.68%)	108.25 ± 46.70^∗∗∗^ (91.27%)

*Note: N* = 4: number of animals; RFE (%), percentage reduction in fecal excretion; values are expressed as mean ± standard error of the mean.

Abbreviations: D, day; HESS, hydroethanolic extract of *S. senegal*.

⁣^∗∗∗^*p* < 0.001 significant difference from negative control.

**Table 3:** Average daily weight gain (ADWG in kilogram) of treated animals.

**Treatments**	**Average daily weight gain posttreatment**			
	**(D**_**22**_ **–D**_**26**_ **)** **(** **n** = 4**)**	**(D**_**22**_ **–D**_**29**_ **)** **(** **n** = 4**)**	**(D**_**22**_ **–D**_**36**_ **)** **(** **n** = 4**)**	**(D**_**22**_ **–D**_**43**_ **)** **(** **n** = 4**)**
Normal control	0.14 ± 0.02	0.21 ± 0.05	0.25 ± 0.05	0.30 ± 0.07
Water	−0.13 ± 0.01	−0.18 ± 0.01	−0.22 ± 0.03	−0.28 ± 0.06
Levamisole	0.08 ± 0.00	0.11 ± 0.00	0.17 ± 0.03	0.23 ± 0.04^∗∗∗^
HESS 125 mg/kg	0.09 ± 0.01	0.30 ± 0.49	0.22 ± 0.04	0.25 ± 0.07^∗∗∗^
HESS 250 mg/kg	0.09 ± 0.01	0.12 ± 0.01	0.19 ± 0.01	0.25 ± 0.04^∗∗∗^
HESS 500 mg/kg	0.03 ± 0.04	0.23 ± 0.01	0.23 ± 0.01	0.29 ± 0.03^∗∗∗^

*Note: N* = 4: number of animals; values are expressed as mean ± standard error of the mean.

Abbreviations: ADWG, average daily weight gain; D, day; HESS, hydroethanolic extract of *S. senegal*.

⁣^∗∗∗^*p* < 0.001: significant difference from negative control.

**Table 4:** Percentage reduction in parasite load (RPL%) in animals treated with different doses of HESS.

**Groups**	**Parasitic load**	**RPL (%)**
Normal control	0.00 ± 0.00	/
Water	835.50 ± 129.60	0.00
Levamisole	81.25 ± 7.63^∗∗∗^	90.27
HESS 125 mg/kg	95.00 ± 6.05^∗∗∗^	88.62
HESS 250 mg/kg	59.00 ± 10.42^∗∗∗^	92.93
HESS 500 mg/kg	44.00 ± 10.16^∗∗∗^	94.73

*Note: N* = 4: number of animals; values are expressed as mean ± standard error of the mean; RPL%: percentage reduction in parasite load.

Abbreviation: HESS, hydroethanolic extract of *S. senegal*.

⁣^∗∗∗^*p* < 0.001: significant difference from negative control.

**Table 5:** The clinical signs observed in mice after administration of HESS.

**Period**	**D**_**1**_				**D**_**2**_	**D**_**3**_	**D**_**4**_	**D**_**5**_	**D**_**6**_	**D**_**7**_	**D**_**8**_	**D**_**9**_	**D**_**10**_	**D**_**11**_	**D**_**12**_	**D**_**13**_	**D**_**14**_
	**1H**	**2H**	**3H**	**4H**													
Grooming	N	N	N	N	N	N	N	N	N	N	N	N	N	N	N	N	N
Convulsion	A	A	A	A	A	A	A	A	A	A	A	A	A	A	A	A	A
Pelage	N	N	N	N	N	N	N	N	N	N	N	N	N	N	N	N	N
Motility	N	N	N	N	N	N	N	N	N	N	N	N	N	N	N	N	N
Stool appearance	N	N	N	N	N	N	N	N	N	N	N	N	N	N	N	N	N
Mortality	A	A	A	A	A	A	A	A	A	A	A	A	A	A	A	A	A

Abbreviations: A, absent; N, normal.

## Data Availability

All data from this work are available from the corresponding author on reasonable request.
